# Age-Related Vestibular Loss: Current Understanding and Future Research Directions

**DOI:** 10.3389/fneur.2016.00231

**Published:** 2016-12-19

**Authors:** Dominic Allen, Luis Ribeiro, Qadeer Arshad, Barry M. Seemungal

**Affiliations:** ^1^Division of Brain Sciences, Imperial College London, London, UK

**Keywords:** vestibular system, aging, vestibular perception, vestibular apparatus, vestibular reflexes

## Abstract

The vestibular system sub-serves a number of reflex and perceptual functions, comprising the peripheral apparatus, the vestibular nerve, the brainstem and cerebellar processing circuits, the thalamic relays, and the vestibular cerebral cortical network. This system provides signals of self-motion, important for gaze and postural control, and signals of traveled distance, for spatial orientation, especially in the dark. Current evidence suggests that certain aspects of this multi-faceted system may deteriorate with age and sometimes with severe consequences, such as falls. Often the deterioration in vestibular functioning relates to how the signal is processed by brain circuits rather than an impairment in the sensory transduction process. We review current data concerning age-related changes in the vestibular system, and how this may be important for clinicians dealing with balance disorders.

## Introduction

Age-related vestibular dysfunction and associated imbalance has a major impact on morbidity, mortality, and health-care resources. According to the National Institute of Deafness and Other Communication Disorders of the NIH, falls account for over 50% of all accidental deaths in the elderly (1), and a recent analysis calculated the medical costs associated with fatal and non-fatal falls in the USA to be over $19 billion annually (2).

The overall prevalence of vestibular dysfunction in adults aged over 40 in the USA is 35.4%, corresponding to 69 million individuals (3). Patients with vestibular dysfunction are at significantly greater risk of falls (odds ratio 12.3 for patients with concurrent dizziness). Although this is also associated with an increased risk of patient-reported dizziness, as many as 32% of individuals aged over 40 without any symptoms of dizziness have evidence of vestibular dysfunction. These patients, though asymptomatic, also have an increased risk of falls (odds ratio 6.3) (3). A case–control study in the UK, in a sample of 56 adults, found that the prevalence of vestibular impairment in older adults who fall was 80%, compared with 19% in age-matched non-fallers (4). Other prospective studies in general practice and neurology clinics have reported that in patients aged over 50 with dizziness, the prevalence of vestibular causes ranges from 18 to 56% (5, 6). Risk factors for decline in vestibular function include smoking, hypertension, and diabetes but even when these are controlled for the effect of age is far more pronounced (3).

Progressive disequilibrium of aging is a complex, multifactorial condition leading to instability and increased risk of falls (7, 8), with vestibular dysfunction, albeit in combination with other factors (e.g., musculoskeletal and visual impairment), being a key contributor to imbalance (9, 10). One factor in balance dysfunction may be changes in the robustness of peripheral vestibular signaling in the elderly (11). Another factor may be changes in how sensory information is processed by central circuits, as exemplified by a study which found that compared with younger subjects, the elderly favor the use of proprioceptive rather than visual and vestibular cues for postural motor control (12). Overall, there is an age-related decline of peripheral vestibular sensing and the central combination of different sensory signals for balance. Herewith, we review the literature regarding these two aspects.

## The Peripheral Vestibular System

Neuronal and hair cell loss are the two biggest effects that aging has on the peripheral vestibular system; affecting both the otolith organs and the semicircular canals. Multiple studies have shown that aging reduces the number of sensory hair cells in the vestibular end organs (13–16). More recently, one group has studied human temporal bone sections from 67 subjects aged from birth to 100 years of age and found that there was a significant age-related decline in the number of hair cells and a decline in individual hair cell subtypes (1, 17).

Several studies have shown degeneration of the vestibular ganglion (Scarpa’s ganglion) and nerve (15, 18–20). The vestibular nerve has two divisions, receiving conveying afferents from both the semicircular canals and the otolith organs *via* the superior and inferior vestibular nerves, respectively (15, 21). Ganglion cell counts from 106 temporal bones from 75 individuals showed age-related reduction in ganglion cell counts with a greater decline in the superior division compared with the inferior division.

### Otolith Organs

The signal from the otolith organs (comprising the utricle and saccule) transduces linear acceleration (and detect tilt), and with respect to these organs, with age, they are not only affected by degeneration of the ganglion cells but also by hair cell loss, in addition to specific degenerative effects within the otolith organ ultrastructure. The use of vestibular evoked myogenic potentials (VEMPs) has been used in multiple studies to assess the effect of aging upon otolith function. VEMPs are short-latency myogenic potentials that are elicited from specific muscles, in response to vestibular stimulation (*via* sound). The muscle measured is the sternocleidomastoid (“cervical” VEMP—cVEMP), hence assessing saccular and inferior vestibular nerve function as well as the inferior oblique muscle of the eye (“ocular” VEMP—oVEMP), which measures utricular and superior vestibular nerve function (22–24). Reduction in the amplitude of VEMPs is indicative of reduced otolith organ function, while increased VEMP latency may relate to slowed brainstem signal processing (25, 26).

Brantberg et al. found an age-related decrease in cVEMP amplitude and increase in cVEMP latency in a study of 1,000 patients ranging from 7 to 91 years old with no known vestibular disorders (25). These findings have been corroborated by Agrawal et al. who found reduced cVEMP as well as oVEMP amplitude in a group of 50 patients above 70 years of age compared to younger individuals (27). Other studies measuring oVEMP have reported similar findings including an age-related increase in oVEMP latencies (28, 29). Further, a more recent study by Li et al. in 257 subjects demonstrated that with aging, there was a reduction in oVEMP amplitude by 2.9 µV per decade of life and an increase in latency of 0.12 ms per decade of life. With respect to cVEMP, they found that the amplitude decreased by 0.14 µV per decade but found no significant difference in latency between the age groups (30).

The otoconia contained in the utricle and saccule have also been shown to undergo morphological changes and degeneration during a human’s lifespan as observed in postmortem analyses (31). Aging has been associated with reduction in otoconia mass as well as fracture and fragment formation in both animals and humans (31–35). While it is easy to assume that reduction in otoconia would result in the reduction of organ function, otoconia degeneration has been shown to affect the utricle more than the saccule (31, 36), which would not explain the findings in the Agrawal et al. study (27). In contrast, it has been previously reported that, while hair loss occurs in all the peripheral vestibular organs with increasing age, the utricle is relatively spared (17). Currently, the implications of otoconia degeneration of otolith organ function are unknown, but it is suspected that these changes in otoconia are involved in the development of peripheral vestibular disorders, such as benign paroxysmal positional vertigo (BPPV) (37).

Benign paroxysmal positional vertigo is one of the most common causes of vertigo, especially in the elderly as there is an increase in the incidence with age, peaking at 60 (38–40). It is a disorder characterized by vertigo upon certain positional head movements. BPPV is caused by the presence of otoconia debris, which moves in the endolymph or cupula of the semicircular canals (41, 42). It is thought that the otoconia are dislodged from the utricular macula, which is precipitated by the morphological changes that can happen to the otoconia during aging (37). While BPPV can be effectively treated with repositioning maneuvers (43), a large observational study of 1,092 BPPV sufferers has recently shown that comorbidities, such as hypertension, osteoporosis, and diabetes, may be correlated with the risk of recurrence of BPPV in the elderly (44).

### Semicircular Canals

The semicircular canals transduce head angular acceleration *via* the anterior, posterior, and horizontal semicircular canals. Decline in the semicircular canals forms a significant component of the overall age-related decline in the vestibular system. A study of 67 human temporal bones from birth to age 100 found that Type I hair cells in the cristae are lost at a significantly greater rate than in the macula (1), further reflected by a cross-sectional study, which found the decline in semicircular canal function to be greater than the decline in otolith function (27). This age-related decline stands in contrast to what happens in peripheral vestibular dysfunction, such as Meniere’s disease, in which there is selective loss of Type II hair cells (45). Decline in the semicircular canals can be evaluated through the angular vestibule–ocular reflex (VOR), for example, using caloric testing; although this technique only tests the horizontal semicircular canals. Up to a few years ago, the only way to assess the VOR was with rotating chairs or by caloric ear stimulation. Recently, advances in understanding of vestibulo–ocular physiology, largely by Curthoys and Halmagyi in Sydney, have led to the development of, first, a bed-side clinical head thrust or impulse test (HIT) and, subsequently, video-image-based versions of the test that are now available commercially for clinical use (vHIT or videoHIT), which allow not only for the assessment of the horizontal but also the anterior and posterior semicircular canals (46).

Numerous studies have investigated age-related decline in the semicircular canal function. Baloh et al. followed 7 patients with severe bilateral vestibulopathy and 51 normal controls over a 5-year period; in the normal subjects, there was a significant decrease in gain and time constant and increase in phase lead of the VOR over this period. Notably, this decline was not associated with any symptoms or signs of disequilibrium (47). By contrast, the patients, whose VOR responses were depressed at the start of testing, did not show any significant decline (48). Carol et al. analyzed 109 subjects using data from the Baltimore Longitudinal Study of Aging and found that VOR gain remained stable from ages 26 to 79, after which it significantly declined at a rate of 0.012/year; the prevalence of VOR gain less than 0.8 was 13% in individuals aged ≥80 compared with 2.8% in those aged under 80 (48).

Agrawal et al. carried out head thrust dynamic visual acuity testing on 50 individuals aged ≥70, finding a significant decline in dynamic visual acuity during tests of all three semicircular canals. Decline in each semicircular canal was strongly correlated with decline in the other two; interestingly, decline in the horizontal and superior semicircular canals was well correlated with decline in utricular but not in saccular function. Decline in posterior semicircular canal function, however, showed no clear trend compared with function of the otolith organs. It was also found that the prevalence of vestibular dysfunction was significantly higher for the semicircular canals (82–94%) compared with the saccule (54–62%) and the utricle (18–24%) (27).

From reviewing the above studies, it can be observed that decline in the function of the semicircular canals plays a significant component of age-related decline in the vestibular system, with a significantly higher prevalence and severity than otolith associated age-related decline. Given the function of the semicircular canals is to measure angular acceleration, it could be postulated that decline in these structures may be more associated with patient-reported dizziness—the presence of which represents a significant increase in the risk of falls in patients with vestibular dysfunction (3).

## The Central Vestibular System

### The Brainstem and Cerebellum

The main component of the brainstem vestibular system is the vestibular nuclear complex straddling the pontomedullary junction. This complex of nuclei receives primary vestibular afferents conveyed by the vestibular nerve and also connects to various structures, including the cerebellum (49). The main vestibular nuclei comprise the descending or inferior (DVN), lateral (LVN), superior (SVN), and medial (MVN) vestibular nuclei (49). Lopez et al., in a study of 15 vestibular nuclei from people aged 40–93, found a neuronal loss of 3% per decade in the vestibular nuclear complex (50, 51). They also found that neuronal loss was higher in the SVN and least in the MVN. This is in contrast to a more recent study of eight brainstems, which showed neuronal loss in the DVN, MVN, and LVN, but sparing of the SVN. This study also found that aging had no effect on the volume or length of the vestibular nuclei (49). However, both studies have found an increase in giant neurons in the elderly, related to lipofuscin deposits within the cells (49, 50). Similar studies have been done in animals, with one study showing an age-related decline in the number of neurons of the mouse vestibular nuclei (52). Conversely, a study in male golden hamsters found conflicting results (53).

The cerebellum plays a critical role in the function of the vestibular system and is known to receive efferent inputs from the vestibular nuclei (54, 55). In aging, cerebellar volume and Purkinje cell density in the cerebellar vermis and white matter in the floccular nodular lobe have been shown to decrease (56–58). There is also a vast network in the cerebral cortex that activates with vestibular stimulation (59–61). Cyran et al. have recently used functional magnetic resonance imaging on 45 subjects aged 20–70 to determine age-related effects on functional connectivity of this vestibular cortical network (62). Using galvanic vestibular stimulation (GVS), which bypasses the peripheral vestibular system and directly stimulates the vestibular nerve, they found a reduction in connectivity with increasing age while controlling for vascular, atrophic, or structural connectivity changes. Jahn et al. have also used GVS to study age-related vestibular function changes in 57 subjects aged 20–69 (63). Specifically, by measuring torsional nystagmus in response to GVS, they found a U-shaped distribution of central vestibular function by age. They speculate that due to a reduction in neuronal hair cells and other peripheral vestibular changes, central processing becomes hypersensitive in order to compensate for such a loss. After the sixth decade, central compensation will breakdown as well and thus lead to impaired vestibular function in the elderly.

The cerebellum is also involved in vestibular adaptation. Previous work has focused on the cerebellar role in VOR adaptation (64). However, recent work has demonstrated an additional but critical role for the cerebellum, which mediates the partitioning of vestibular signals involved in eye movement control versus those that ascend to perceptual regions mediating sensations of self-motion (i.e., vertigo) and spatial orientation (65). Curiously, relatively little work has been focused on the effect of aging upon cerebellar function (66). However, it is likely that aging in the cerebellum will impact directly upon vestibular reflex and perceptual functioning and adaptation to lesions or with training.

## The Vestibular Thalamic Projections and the Vestibular Cortical System

Spatial orientation is a critically important function in everyday life. Up to third of newly diagnosed dementia patients complain of spatial disorientation (67), causing significant disruption of everyday life. A core brain area implicated in spatial orientation and memory is the hippocampus (67). Indeed, previous neuroimaging study has shown hippocampal atrophy with bilateral vestibular failure (68). Animal neuronal recordings also show cells sensitive to spatial orientation status that are disrupted by vestibular loss. A key concept is the notion of converting vestibular motion signals to spatial signals. Given the above evidence, it has been argued that the hippocampus is important for this. However, some authors have found normal path integration function with hippocampal lesions in humans but not rats (69). This conundrum has recently been solved by a recent human lesion study, which shows in fact that the important region is the temporoparietal junction (70). In addition, this study also found no impact of hippocampal lesions upon angular path integration function. It follows that dementia, which is more frequent in the elderly, may affect spatial orientation by its effect on vestibular cortical regions such as the TPJ (70).

Another currently unsolved question is the cortical location mediating the sensation of vertigo. Current wisdom suggests that the posterior insular cortex is the primary vestibular cortex. However, focal stroke, including in the posterior insular, did not affect vestibular sensation of self-motion (kaski). Previous work (65, 71) suggests, however, that the vestibular sensation of self-motion may be distributed and hence not localizable. Whether such vestibular cortical networks are disrupted by aging will require further work.

## Conclusion

As with most systems in the body, aging causes a degenerative effect within the vestibular system. Aging in the vestibular system is a multifactorial process, affecting both the peripheral organ and central circuits, from the peripheral end-organ to the brainstem to the cerebellum to the cerebral cortex. It follows that diseases that affect any one of these brain areas will disrupt one or more facets of vestibular functioning. Recent studies using VEMP and VOR testing have shown that there is a quantifiable decline in function in specific peripheral vestibular organs with age, which theoretically correlates with the histological and microscopic changes previously seen. There is also similar ongoing research using GVS to identify functional loss with age of central vestibular pathways. While the cause of dizziness in the elderly is a multisystem processes, the data suggest that aging causes a reduction in peripheral vestibular function and also the cortical efficiency with which these signals are used for balance, which together play a significant role in the increasing the risk of falls in the elderly.

## Author Contributions

LR and DA: initial drafting of manuscript. QA: initial drafting and final revision of manuscript. BS: general organization of manuscript. Interim and final revision of manuscript.

## Conflict of Interest Statement

The authors declare that the research was conducted in the absence of any commercial or financial relationships that could be construed as a potential conflict of interest. The reviewer MS and handling Editor declared their shared affiliation, and the handling Editor states that the process nevertheless met the standards of a fair and objective review.
