# Corrigendum: Age-Related Vestibular Loss: Current Understanding and Future Research Directions

**DOI:** 10.3389/fneur.2017.00391

**Published:** 2017-08-21

**Authors:** Dominic Allen, Luis Ribeiro, Qadeer Arshad, Barry M. Seemungal

**Affiliations:** ^1^Division of Brain Sciences, Imperial College London, London, United Kingdom

**Keywords:** vestibular system, aging, vestibular perception, vestibular apparatus, vestibular reflexes

**Dominic Allen** and **Luis Ribeiro** were not included as authors in the published article. The authors apologize for this error and state that this does not change the scientific conclusions of the article in any way.

The original article has been updated.

## Author Contributions

LR and DA: initial drafting of manuscript. QA: initial drafting and final revision of manuscript. BS: general organization of manuscript. Interim and final revision of manuscript.

## Conflict of Interest Statement

The authors declare that the research was conducted in the absence of any commercial or financial relationships that could be construed as a potential conflict of interest.

